# Keratoacanthoma and Keratoacanthoma-Like Squamous Cell Carcinoma: Similar Morphology but Different Pathogenesis

**DOI:** 10.1097/MD.0000000000000934

**Published:** 2015-06-12

**Authors:** Isabela C. Watanabe, Renata F. Magalhães, Aparecida M. de Moraes, Rafael F. Stelini, Geórgia F. Cintra, Konradin Metze, Maria L. Cintra

**Affiliations:** From the Department of Pathology (ICW, RFS, GFC, KM, MLC); Department of Dermatology (RFM, AMM), Medical Sciences School, State University of Campinas, Campinas, Brazil.

## Abstract

Differential diagnosis between keratoacanthoma (KA) and squamous cell carcinoma (SCC) is difficult due to their similarities. The mechanisms that drive their distinct biological behavior are poorly understood.

To investigate whether the assessment of microvessel density (MVD) could be helpful in KA and SCC differential diagnosis and to gain insight into the pathogenesis of KA-like neoplasms, we compared the density of CD105- and CD34-stained vessels in KAs and SCCs and their relation to the expression of the p53 oncoprotein and proliferation marker Ki67.

This is an observational retrospective cohort study. Forty lesions with clinical appearance of KAs (29 KAs and 11 SCCs) entered the study. A biopsy was taken from each lesion at presentation and the natural clinical course was monitored for at least 1 month. Growing or minimally regressing lesions were submitted to complete surgical excision. The diagnoses were established on combined clinical, histological, and follow-up evaluations. The MVD and p53 or Ki67 expression in neoplastic cells were assessed through morphometry.

The MVD did not show discriminating power between KAs and SCCs. The Ki67 proliferation rate was significantly higher in SCCs. Although neoangiogenesis (CD105-MVD) in KAs was associated with cell proliferation, in SCCs it was not. There was significant correlation between p53 expression and neoplasia size in SCCs but not in KAs.

From our results, we may conclude that KA and SCC have similarities, as CD105- and CD34-MVD. However, the low Ki67 proliferation index and the positive correlation between Ki-67 index and neovascularization in KA suggest a dependence in neovascularization to grow in KA, pointing to involvement of distinct pathogenesis.

## INTRODUCTION

### Background

Keratoacanthoma (KA) is a cutaneous neoplasia arising preferably from hair follicle cells on sun-exposed skin and characterized by self-limiting growth and involution. The life cycle from origin to spontaneous resolution takes about 4 to 6 months in the majority of cases^1^ and consists of 3 distinct stages: proliferative, mature, and involutional. The real nature of this neoplasma and its relationship to squamous cell carcinoma (SCC) have been disputed for a long time. Some authors consider KA as a benign neoplasia, others as a precursor of SCC,^2^ others as a well-differentiated variant of cutaneous SCCs ^3^ or at least an abortive malignancy that only rarely progresses into an invasive SCC.^4^ Differential diagnosis between KA and SCC is challenging due to their similarities and the lack of reliable diagnostic criteria to distinguish both entities.^5^

Several studies have attempted to find more specific methods to distinguish KAs and SCCs accurately. Among them, Stephenson et al^6^ studied the expression of p53 protein in KAs and SCCs and found no clear-cut distinction between these neoplasms. Kerschmann et al^7^ analyzed the expression of p53 tumor suppressor protein and the proliferation index (Ki67) in KAs and SCCs and found that both neoplasms contain stainable quantities of p53 protein suggesting that these neoplasms are best viewed as a spectrum. These authors also did not find a statistically significant difference in proliferation rate between neoplasms that express p53 and those that do not, for either KA or SCC.

Kaabipour et al^8^ studied the expression of p16, a tumor suppressor protein, in KAs and SCCs and found no significant differences that could make this marker useful to this differential.

Kannon et al^9^ found that the expression of a peanut lectin receptor is usually uniformly positive in KAs and negative in the majority of SCCs, indicating that this could aid in the histologic differentiation of these neoplasms. Takeda et al^10^ demonstrated differences in the expression of angiotensin type 1 receptors between SCCs and KAs. However, both of these methods are neither definitive nor reliable to differentiate KAs and SCCs in all cases.

The study of the microenvironment in neoplasia growth, invasion, and metastatic potential is of increasing interest because its stimulatory and restrictive effects upon neoplasm progression have become evident.^11–13^ In fact, previous works have shown that the neoplastic microenvironment plays a major role in several phases of neoplasm development.^14,15^ Takahara et al^16^ studied the microenvironment of epidermal neoplasms and suggested that increased macrophages and decreased Langerhans cells are associated with proliferation and invasion of malignant epidermal tumors. These authors found a significant higher number of dermal macrophages, which are thought to be associated with angiogenesis in SCC, compared with KA and other epidermal tumors.

Induction and remodeling of angiogenesis have been pointed out to be relevant for tumor progression and malignancy.^11–13^ In fact, angiogenesis has been reported to be an important prognostic factor of outcome for several malignancies.^15,17,18,^ Differences in microvessel density (MVD) could also be related to the distinct behavior between KA and SCC and help distinguish between these entities. Weninger et al^19^, however, could not find a significant difference in factor VIII-determined MVD comparing KAs and SCCs.

CD105 (endoglin) is an 180-kDa homodimeric transmembrane protein overexpressed in proliferating endothelial cells.^20^ The observations that CD105 was strongly up-regulated in the endothelium within various tumors compared with normal tissues supported the important role of CD105 in the investigation of tumor angiogenesis.^21–23^ CD105 has been demonstrated to be an endothelial cell proliferation-associated marker and to represent a powerful indicator of neovascularization.^20^ To the best of our knowledge, CD105-determined MVD has not been studied in KAs.

### Hypotheses and Specific Objectives

To investigate whether the assessment of MVD could be helpful in KA and SCC differential diagnosis and to gain insight into the pathogenesis of KA-like neoplasms, we compared the density of CD105 and CD34 stained vessels in KAs and SCCs and their relation to the expression of the p53 oncoprotein and the proliferation marker Ki67.

## METHODS

### Study Design

This is an observational retrospective cohort study.

### Patients and Material

This study has been approved by the Research Ethics Committee of the State University of Campinas (ID number 822/2005).

Routinely, formalin-fixed and paraffin embedded specimens of 43 KA-like lesions from 41 patients were selected from the files of the Department of Pathology, State University of Campinas (UNICAMP). These cases corresponded to the cohort of a previous study ^24^ in which we analyzed the histopathologic features associated with clinical regression in KA-like lesions.

In brief, during a period of 4 years, when a patient with a cutaneous KA-like lesion (firm, dome-shaped, smooth, enlarging papule or dome-shaped nodule with a central umbilicated keratinous core) was referred to the dermatological outpatient center, the following routine was taken by the same dermatologist (RFM): cutaneous examination findings were recorded; photos were taken and a partial biopsy was performed so as to include its center and side throughout its depth, to allow the evaluation of the neoplasia architecture and the bottom neoplasia border. A follow-up appointment was scheduled 1 month later for clinical re-evaluation by the same dermatologist as well as lesion measurement and definition of treatment modality. In cases of growing (progression, infiltration and lack of involution) or minimally regressing lesions, complete surgical excision, as for SCC, was done. In contrast, involuting lesions were left untreated. The patients’ ages ranged from 27 to 93 (mean 71.5) years and the lesions diameter ranged from 5 to 40 (mean 19) mm.

After combined clinical, histological, and follow-up evaluations, 32 of 43 lesions were assessed as KA and 11 as SCC (aggressive course and histological features of SCC). Twelve KAs were partially excised (first biopsy) in the intermediate stage between proliferative and fully developed, and 20 in the intermediate stage between the fully developed and regressing phase. An additional 3 lesions were excluded from the present study because of lack of sufficient material to perform immunostaining.

No lesions with obvious clinical signs of involution at presentation nor special morphologic or syndromic types of KAs were included. No recurrence or metastasis developed after a follow-up of 3 months to 10 years.

### Immunohistochemical Staining

The biopsies of all 40 lesions (29 KAs and 11 SCCs) were formalin-fixed. Sections (4-μm thick) were cut from the retrieved neoplasm blocks, dewaxed, and rehydrated in graded ethanol. The primary antibodies used were either p53 (D07; DakoCytomation, Glostrup, Denmark) at a dilution of 1:100, Ki67 (mib1; Dako) at a dilution of 1:150, or CD34 (NCL-END, Novocastra, Newcastle upon Tyne, UK) at a dilution of 1:100 or CD105 (SN6 h; Dako) at a dilution of 1:10. For all antibodies with the exception of CD105, a steamer was used for epitope retrieval with either citrate buffer (CD34) or Tris-ethylenediaminetetraacetic acid (EDTA) buffer (p53 and Ki67). For CD105, antigen retrieval was performed using 0.4% pepsin at 37 °C for 30 minutes. Only the CD105 sections were incubated at 37 °C with protein block serum-free (Dako). The EnVision Plus polymer (Dako) was used as a reaction amplifier. Visualization of the antibody complex was achieved using 3,3-diaminobenzidine tetrahydrochloride according to the manufacturer's instructions. Sections were counterstained with hematoxylin. Appropriate controls were included in each assay. For negative controls, primary antibodies were omitted and the specimens were incubated with antibody diluent. Tissue from cutaneous SCC with optimal fixation and blocking was used as positive control.

### Microvessel Quantification

The sections were blindly scanned at low magnification (×100) by a single pathologist for areas of highest microvessel density), so-called “hot spots.”^25^ For each section, starting at the area assumed as the “hot spot,” 10 images of consecutive high-power fields were obtained using a E200 Nikon microscope equipped with a A630 Canon camera, both for CD34 and CD105. When the size of the biopsy did not allow the capture of 10 different images, all distinct fields were photographed. Areas along the border between neoplasm and surrounding tissue were preferred, whereas areas next to hair follicles were avoided. Any brown-stained single cells or clusters of cells, which were separated from other stained areas, were considered a single countable vessel. Unstained lumina were not taken into account. For each image, the total stromal area was calculated using computer-assisted image analysis (Imagelab analysis software, 2000), which allows manual segmentation of target areas. The stromal areas measured up to 0.04 mm^2^ in each digital image. MVD was defined as the number of manually counted stained vessels per mm^2^ (v/mm^2^). The MVD of the most vascularized area of each section and the MVD corresponding to the sum of all vessels and stromal areas analyzed per section were calculated (total MVD).

### p53 and Ki67 Quantification

As for the vascular markers, the sections stained with p53 and Ki67 were blindly scanned for “hot spots” and ≤10 images of consecutive high-power fields were obtained according to the size of the biopsy. A quantitative analysis of the expression of each marker was performed using computer-assisted image analysis (Imagelab analysis software, 2000). All positive and negative nuclei of epithelial neoplasm cells were counted in each image. The neoplasm expression of these markers was calculated as the percentage of positive cells in the most positive area as well as the percentage of positivity corresponding to the total amount of nuclei counted per section.

### Statistical Analysis

The data obtained from the incisional biopsies of the 40 KA-like lesions were analyzed (KM and ICW). The CD34 and CD105-determined MVD and the p53 and Ki67 expression percentage were calculated with Winstat 3.1 software. Normal distribution of the variables was tested according to Kolmogorov–Smirnov. Student *t* test was used to evaluate differences between both groups. Correlations among the variables were assessed by Pearson correlation coefficient analysis. In addition, multiple liner regressions were calculated, which enables us to determine whether correlations are independent. Moreover, we calculated the coefficients of determination *R*^2^. The significance level was 0.05.

## RESULTS

The study comprised 29 KAs and 11 SCCs, which had been classified primarily according to clinical follow-up.

**FIGURE 1:**
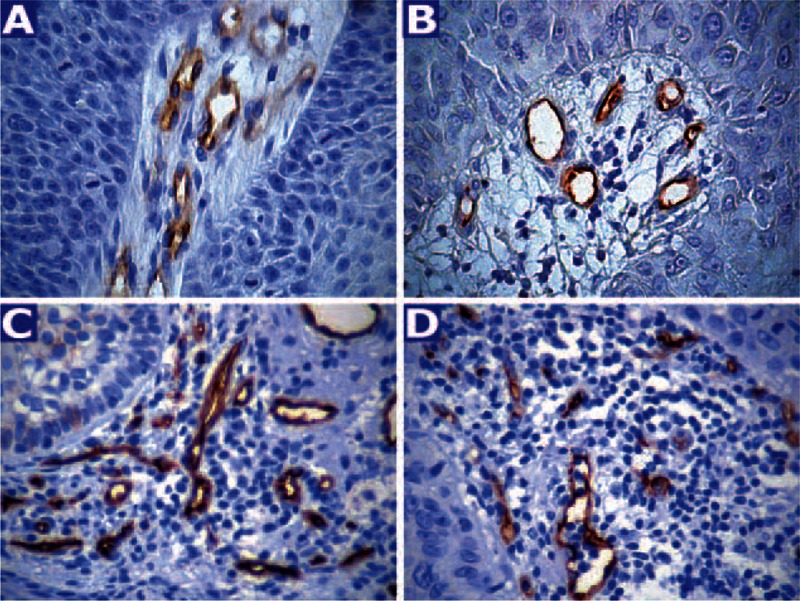
Immunostaining of microvessels using CD105 in KA (A) and SCC (B). CD34 membrane staining of endothelial cells in KA (C) and SCC (D). Original magnification: ×400.

**FIGURE 2:**
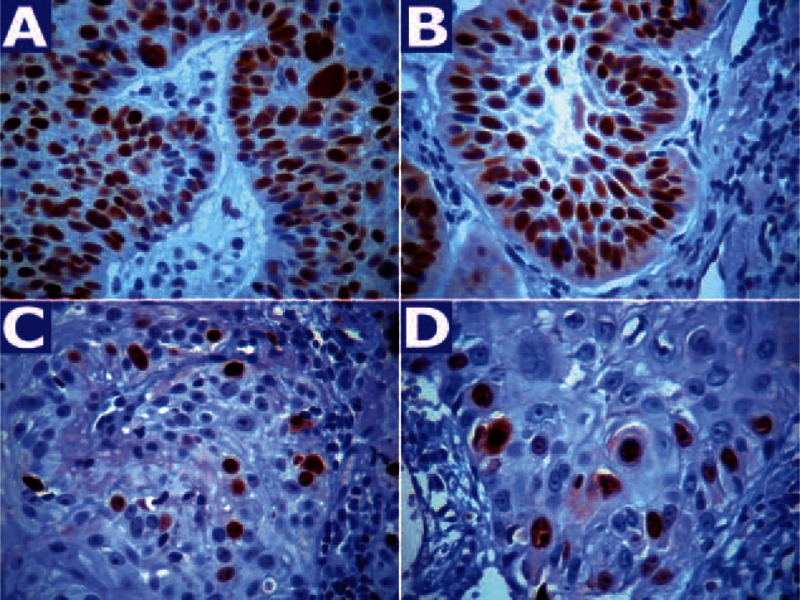
Diffuse nuclear p53 immunostaining in KA (A) and SCC (B); Ki67 immunostaining in KA (C) and SCC (D). Original magnification: ×400.

CD34 and CD105 antibodies provided a uniform and moderate (CD105) to intense (CD34) membrane staining of endothelial cells with no or very low background (Figure 1A-D). Immunohistochemical detection of p53 and Ki67 proteins was characterized by nuclear staining that varied in intensity but was comparable to positive controls (Figure 2A-D). In the Kolmogorov test, all examined variables showed a close approximation to the normal distribution, thus confirming the use of parametric tests.

**FIGURE 3:**
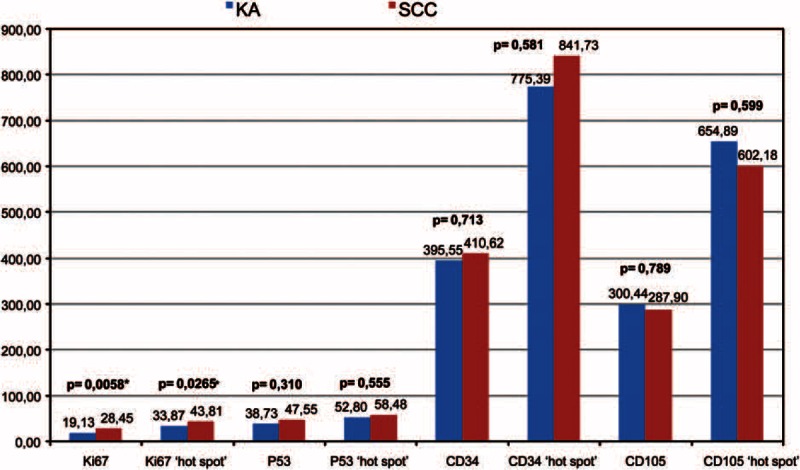
Expression of Ki67, p53, CD34, and CD105 in keratoacanthomas and squamous cell carcinomas. There were no significant differences between KAs and SCCs regarding the CD34 or CD105 microvessel density nor the p53^+^ cells density. The Ki67 staining index was significantly higher in SCCs.

There were no statistically significant differences between KAs and SCCs in microvessel density revealed by CD34 (*P* > 0.2) or CD105 staining (*P* > 0.2), as well as the density of p53 positive nuclei (*P* > 0.2). The Ki67 staining index was significantly higher in SCCs (*P* = 0.0058) (Figure 3). SCCs were also compared with each KA phase by analysis of variance, followed by the least significant difference post-hoc test. Correlations among the variables were assessed by Pearson correlation coefficient analysis. As potential differences between the different KA phases could provoke pseudo correlations, we also calculated partial regressions with the variable “KA phase.” There were significant differences in Ki67 staining index (*P* = 0.005) between the groups. The lowest values were found in the fully developed/regressing KAs, followed by the proliferative/fully developed KAs, and the highest values were found in SCCs. The post-hoc test showed only significant differences between the regressing KAs and SCCs. There was, however, considerable overlap between the values in the 3 groups.

**FIGURE 4:**
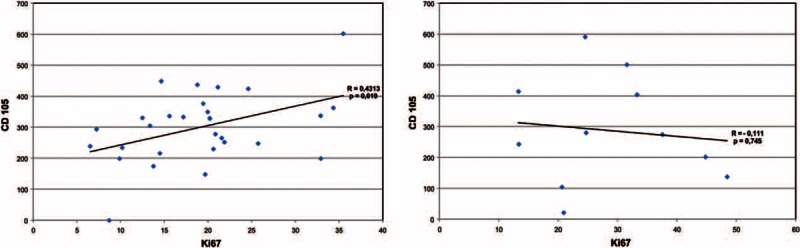
Significant positive correlation of MVD-CD105 and Ki67-proliferation index in KAs (A). This was not the case for SCCs (B).

In SCCs, there were no significant correlations between the density of CD105-positive vessels and age, neoplasm size, Ki67, or p53 index (*P* > 0.2 for all). We found a positive correlation in KAs between the CD105 density and the Ki67 index (*r* = 0.43; *P* = 0.019; Figure 4A). This was not the case for SCCs (Figure 4B). In a multiple linear correlation for CD105-MVD in KAs, the Ki67 index remained as an independent variable (*P* = 0.004; *R*^2^ = 0.31), whereas in multiple linear correlation for SCCs, this result was not found.

**FIGURE 5:**
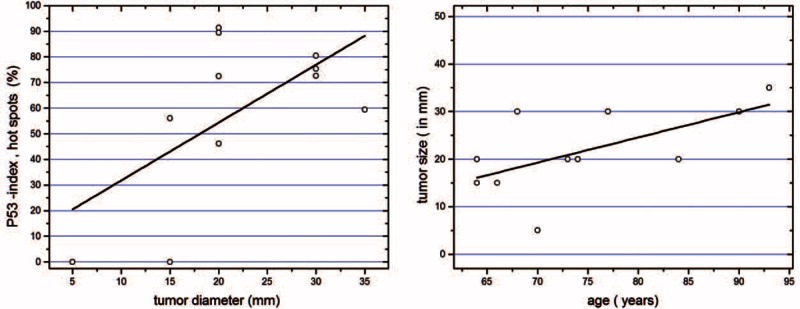
Significant positive correlation between the neoplasma size and p53 index (A) and neoplasm size and age in SCC (B).

We did not find any significant correlation for KAs between the size of the neoplasm and patient's age, Ki67, or p53-index (*P* > 0.2 for all). However, in SCCs, there were significant positive correlations between the neoplasm size and p53 index (*r* = 0.59, *P* = 0.05) and the neoplasm size and age (*r* = 0.617; *P* = 0.043), which demonstrated independence in a multiple linear regression (Figure 5).

## DISCUSSION

KA is a squamous proliferative lesion, which has become a neoplasm of great interest in the study of carcinogenesis because of its unique natural history and controversial relationship to SCC. The reason for the distinct biological behavior between these neoplasms is not yet understood.

Angiogenesis is considered to play a major role in neoplasm progression, as neoplasm growth is dependent on a sufficient supply of nutrients and oxygen.^26^ In contrast to SCC, a malignant tumor with metastatic potential, KA does not metastasize. Weninger et al^19^ investigated MVD in basal cell carcinomas (BCCs), SCCs, and KAs and found that the vascular density was significantly lower in BCC than in both KA and SCC, but no significant difference in MVD was demonstrated between the latter neoplasms. Our results are in accordance with their findings because the assessment of MVD using both a pan-endothelial marker (CD34) and a neovascularization marker (CD105) did not reveal statistical differences between them. We share the opinion of these authors that neoplasm MVD probably does not reflect the neoplasm progression differences between them.

Our quantitative data from the proliferation rate (Ki67) did show discriminating power between KAs and SCCs, with a rate significantly higher in SCCs. However, there was a large overlap between both groups, making it difficult to apply in routine practice because pathologists most commonly use semiquantitative methods of evaluation. Still, is of interest that the SCCs, neoplasm with a higher malignant potential than KAs, had the higher proliferation rate. With respect to the time of neoplasm growth, it is known that KAs generally grow faster than most SCCs, which seems to conflict with the proliferation rate found by our study. This can be explained by the fact that part of our KAs was in the intermediate stage between the fully-developed and regressing phases. Indeed, when SCCs were also compared with each KA phase by the post hoc test, significant differences were only found between the regressing KAs and SCCs. Our other quantitative data did not show discriminating power between KAs and SCCs, adding to the hall of similarities previously described for these 2 neoplasms.

The immunostains assessed in our study demonstrated important differences regarding neoplasm pathogenesis. Although neoangiogenesis (CD105-MVD) in KAs was associated with neoplasm proliferation, in SCCs, it was not. It is probable that KA needs neovascularization to grow and SCCs have independent growth, probably due to genetic differences. Waring et al^27^ found rarity of loss of heterozygosity in KAs, in contrast with what was described for SCCs. In Clausen et al’^28^ study, KA showed a surprisingly high degree of genetic imbalance. Many of the most frequently found aberrations in KAs were not seen in any of the SCCs analyzed by the authors and could be linked to their peculiar rapid growth followed by regression.

In addition, the correlations observed between age and neoplasm size or p53 expression and neoplasm size in SCCs but not in KAs also indicate further different mechanisms of progression for each neoplasm.

We consider that the strengths of this study are as follows: the diagnoses of SCC and KA were established on clinical/pathological/follow-up basis; the lesions were relatively uniform in size and the patients’ ages relatively uniform, in each group. To the best of our knowledge, this is the first systematic study on angiogenesis of lesions with clinical appearance of KA in which, to improve the histopathologic criteria that discriminate the 2 neoplasms, clinical evolution was used as gold standard. However, the small sample size for KA-like SCCs may have restricted the test power for this group and is a limitation of our study.

In summary, from our results, we may conclude that KA and SCC have similarities, as CD105- and CD34-MVD. However, the low Ki67 proliferation index and the positive correlation between Ki-67 index and neovascularization on KA suggest dependence in neovascularization to grow in KA, pointing to involvement of distinct pathogenesis.

## References

[1] Schwartz RA Keratoacanthoma. *J Am Acad Dermatol* 1994; 30:1–19. 8277007 10.1016/s0190-9622(94)70001-x

[2] Strieth S Hartschuh W Pilz L Carcinoma-like vascular density in atypic keratoacanthoma suggests malignant progression. *Br J Cancer* 2002; 87:1301–1307. 12439721 10.1038/sj.bjc.6600622 PMC2408913

[3] Hodak E Jones RE Ackerman AB Solitary keratoacanthoma is a squamous-cell carcinoma: three examples with metastases. *Am J Dermatopathol* 1993; 15:332–342. 8214391 10.1097/00000372-199308000-00007

[4] Manstein CH Frauenhoffer CJ Besden JE Keratoacanthoma: is it a real entity? *Ann Plast Surg* 1998; 40:469–472. 9600429 10.1097/00000637-199805000-00004

[5] Schwartz RA The keratoacanthoma: a review. *J Surg Oncol* 1979; 12:305–317. 392196 10.1002/jso.2930120404

[6] Stephenson TJ Royds J Silcocks PB Mutant p53 oncogene expression in keratoacanthoma and squamous cell carcinoma. *Br J Dermatol* 1992; 127:566–570. 1476915 10.1111/j.1365-2133.1992.tb14866.x

[7] Kerschmann RL McCalmont TH LeBoit PE p53 oncoprotein expression and proliferation index in keratoacanthoma and squamous cell carcinoma. *Arch Dermatol* 1994; 130:181–186. 8304756

[8] Kaabipour E Haupt HM Stern JB p16 expression in keratoacanthomas and squamous cell carcinomas of the skin. An immunohistochemical study. *Arch Pathol Lab Med* 2006; 130:69–73. 16390241 10.5858/2006-130-69-PEIKAS

[9] Kannon G Park HK Utility of peanut agglutinin in the diagnosis of squamous cell carcinoma and keratoacanthoma. *Am J Dermatopathol* 1990; 12:31–36. 2180338 10.1097/00000372-199002000-00005

[10] Takeda H Kondo S Differences between squamous cell carcinoma and keratoacanthoma in angiotensin type-1 receptor expression. *Am J Pathol* 2001; 158:1633–1637. 11337361 10.1016/S0002-9440(10)64119-3 PMC1891940

[11] Sharma S Sharma MC Sarkar C Morphology of angiogenesis in human cancer: a conceptual overview, histoprognostic perspective and significance of neoangiogenesis. *Histopathology* 2005; 46:481–489. 15842629 10.1111/j.1365-2559.2005.02142.x

[12] Vermeulen PB Gasparini G Fox SB Second international consensus on the methodology and criteria of evaluation of angiogenesis quantification in solid human tumors. *Eur J Cancer* 2002; 38:1564–1579. 12142044 10.1016/s0959-8049(02)00094-1

[13] Offersen BV Borre M Overgaard J Quantification of angiogenesis as a prognostic marker in human carcinomas: a critical evaluation of histopathological methods for estimation of vascular density. *Eur J Cancer* 2003; 39:881–890. 12706356 10.1016/s0959-8049(02)00663-9

[14] Soares AB Juliano PB Araujo VC Angiogenic switch during tumor progression of carcinoma ex-pleomorphic adenoma. *Virchows Arch* 2007; 451:65–71. 17593387 10.1007/s00428-007-0438-z

[15] Weidner N Folkman J Pozza F Tumor angiogenesis: a new significant and independent prognostic indicator in early-stage breast carcinoma. *J Natl Cancer Inst* 1992; 84:1875–1887. 1281237 10.1093/jnci/84.24.1875

[16] Takahara M Chen S Kido M Stromal CD10 expression, as well as increased dermal macrophages and decreased Langerhans cells, are associated with malignant transformation of keratinocytes. *J Cutan Pathol* 2009; 36:668–674. 19515046 10.1111/j.1600-0560.2008.01139.x

[17] Srivastava A Laidler P Davies RP The prognostic significance of tumor vascularity in intermediate-thickness (0.76-4.0 mm thick) skin melanoma. A quantitative histologic study. *Am J Pathol* 1988; 133:419–423. 3189515 PMC1880778

[18] Weidner N Carroll PR Flax J Tumor angiogenesis correlates with metastasis in invasive prostate cancer. *Am J Pathol* 1993; 143:401–409. 7688183 PMC1887042

[19] Weninger W Rendl M Pammer J Differences in tumor microvessel density between squamous cell carcinomas and basal cell carcinomas may relate to their different biologic behavior. *J Cutan Pathol* 1997; 24:364–369. 9243364 10.1111/j.1600-0560.1997.tb00805.x

[20] Fonsatti E Sigalotti L Arslan P Emerging role of endoglin (CD105) as a marker of angiogenesis with clinical potential in human malignancies. *Curr Cancer Drug Targets* 2003; 3:427–432. 14683500 10.2174/1568009033481741

[21] Wang JM Kumar S Pye D A monoclonal antibody detects heterogeneity in vascular endothelium of tumours and normal tissues. *Int J Cancer* 1993; l54:363–370. 8509210 10.1002/ijc.2910540303

[22] Burrows FJ Derbyshire EJ Tazzari PL Up-regulation of endoglin on vascular endothelial cells in human solid tumors: implications for diagnosis and therapy. *Clin Cancer Res* 1995; 1:1623–1634. 9815965

[23] Kumar P Wang JM Bernabéu C CD105 and angiogenesis. *J Pathol* 1996; 178:363–366. 8691311 10.1002/(SICI)1096-9896(199604)178:4<363::AID-PATH491>3.0.CO;2-8

[24] Magalhães RF Cruvinel GT Cintra GF Diagnosis and follow-up of keratoacanthoma-like lesions: clinical and histologic study of 43 cases. *J Cutan Med Surg* 2008; 12:163–173. 18627696 10.2310/7750.2008.07042

[25] Weidner N Semple JP Welch WR Tumor angiogenesis and metastasis- correlation in invasive breast carcinoma. *N Engl J Med* 1991; 324:1–8. 1701519 10.1056/NEJM199101033240101

[26] Folkman J What is the evidence that tumors are angiogenesis dependent? *J Natl Cancer Inst* 1990; 82:4–6. 1688381 10.1093/jnci/82.1.4

[27] Waring AJ Takata M Rehman I Loss of heterozygosity analysis of keratoacanthoma reveals multiple differences from cutaneous squamous cell carcinoma. *Br J Cancer* 1996; 73:649–653. 8605102 10.1038/bjc.1996.113 PMC2074334

[28] Clausen OP Beigi M Bolund L Keratoacanthomas frequently show chromosomal aberrations as assessed by comparative genomic hybridization. *J Invest Dermatol* 2002; 119:1367–1372. 12485441 10.1046/j.1523-1747.2002.19613.x

